# Techniques for the collection, transportation, and isolation of orchid endophytes from afar: a case study from Madagascar

**DOI:** 10.1186/s40529-017-0209-3

**Published:** 2017-11-28

**Authors:** Lawrence W. Zettler, Landy Rajaovelona, Kazutomo Yokoya, Jonathan P. Kendon, Andrew L. Stice, Amanda E. Wood, Viswambharan Sarasan

**Affiliations:** 1 Department of Biology, Illinois College, 1101 West College Ave., Jacksonville, IL 62650 USA; 2 Kew Madagascar Conservation Centre, Lot #J131B, Ambodivaonjo-Ivandry, 101, Antananarivo, Madagascar; 3 Royal Botanic Gardens Kew, Richmond, Surrey, TW9 3AB UK; 4 Environmental Studies Program, University of Pennsylvania, Philadelphia, PA 19104 USA

**Keywords:** Orchidaceae, Conservation, Epiphytes, *Tulasnella*, *Ceratobasidium*

## Abstract

**Background:**

Tropical orchids need more study with respect to their mycorrhizal associations. For researchers in distant countries who aspire to study these orchids augmenting their conservation, the great distances involved, coupled with limited funds, pose formidable challenges. These challenges are sometimes exacerbated by political unrest, delays in securing permits, unexpected hardships, and the risk that the biological samples collected (e.g., roots harboring mycorrhizal fungi) will not survive long-distance transport.

**Results:**

We describe a protocol for the collection and transport of root samples from Madagascar orchids to labs in the United Kingdom (Kew) and the United States (Illinois) where *Rhizoctonia*-like fungi were subsequently isolated. Three separate trips were made spanning 4 years (2012–2015), with emphasis on the collection of roots from epiphytic, lithophytic, and terrestrial orchids inhabiting the Itremo Massif of the Central Highlands. Collectively, the trips to Madagascar resulted in the isolation of all major groups of *Rhizoctonia*-like fungi (*Ceratobasidium*, *Tulasnella*, *Sebacina*) from all three orchid growth forms (terrestrials, epiphytes and lithophytes). Sampling of terrestrial and epiphytes during the rainy season (January) yielded best results.

**Conclusions:**

Our study demonstrates that peloton-forming fungi in root samples can retain viability up to 3 weeks after collection.

## Background

Orchids have the unparalleled distinction of being the most diverse plant family on earth, but also the most vulnerable to extinction (Swarts and Dixon 2009; Merritt et al. 2014). Part of their vulnerability stems from the family’s susceptibility to acute changes in their environment exacerbated by climate change, as well as their extreme dependence on pollinators and mycorrhizal fungi to complete their life cycles. Of the approximately 25,000 species of orchids worldwide (Dressler 1993; Cribb et al. 2003), about two-thirds are represented by epiphytes and lithophytes (Swarts and Dixon 2009), the majority of which are confined to tropical latitudes in areas prone to deforestation. The remaining one-third consist of terrestrials, many of which occupy cooler temperate zones that are undergoing rapid warming. Collectively, orchids face a conservation crisis of epic proportions, and understanding their biotic and abiotic needs is crucial to their survival.

Mycorrhizal fungi are needed by orchids as a carbon source to initiate germination of their dust-like seeds (Rasmussen 1995). Most of these fungi fall under the category of basidiomycetes in the *Rhizoctonia* complex (Currah et al. 1997)—a group that persists mostly as free-living saprophytes (Moore-Landecker 1996), but also pathogens, microparasites, and orchid symbionts (Swarts and Dixon 2017). Unlike other mycorrhizal associations throughout the plant kingdom, orchids exploit their fungi as a food source (mycotrophy) while providing no substantial benefits in return (Rasmussen and Rasmussen 2009). At maturity, terrestrial and epiphytic orchids alike are thought to maintain a lifelong association with fungi, at least to some extent, providing these plants with ‘trophic versatility’, i.e., a dual mechanism that utilizes both photosynthesis and mycotrophy. The importance of mycorrhizal fungi, therefore, spans the life of the orchid, and conserving these unique plants requires that we also understand and conserve their fungal associates. To effectively do so, we must understand how orchids interact with fungi in the natural setting, particularly at the germination site, exemplified by recent studies (e.g., Jacquemyn et al. 2014; McCormick et al. 2012; Swarts et al. 2010). Mycorrhizal fungi must also be isolated from living orchid tissues, identified, screened for their ability to germinate seeds in vitro verifying their functionality, and safeguarded in cryopreservation.

During the past 30+ years, much has been published on orchid endophytes recovered from temperate terrestrials (e.g., Currah et al. 1987; Warcup 1981; Zelmer et al. 1996), and more recently the tropical epiphytes (e.g., Pereira et al. 2003; Nontachaiyapoom et al. 2010; Chen et al. 2012; Hoang et al. 2017), but the lithophytes remain in need of more study. The length of time that fungi can remain viable in orchid tissues from field collection to the lab is also unclear. Swarts and Dixon (2017) recommended that peloton extraction take place the same day of root collection, and indeed many studies have adopted this protocol (e.g., Aggarwal et al. 2012; Chutima et al. 2011; Otero et al. 2002; Pereira et al. 2005), as it is generally assumed that fungal pelotons lose their viability shortly after root detachment. Suárez et al. (2006), for example, reported that hyphae in Andean orchid tissues lose viability even after one night of storage in the laboratory regardless of chilling. Nevertheless, samples may be processed 3–4 days after collection if necessary (Swarts and Dixon 2017), or longer in some cases. Zettler et al. 2011, for example, recovered orchid endophytes from root samples that were collected 1-week prior. Similarly, Richardson et al. (1993) had success in isolating a diverse assemblage of *Rhizoctonia*-like fungi from epiphytes in Costa Rica up to 3 weeks after collection.

In Madagascar, where 90% of the island’s 1000 orchid species are endemic (Tyson 2000), deforestation on a massive scale has resulted in patches of fragmented orchid populations that persist from year to year with little natural regeneration (Whitman et al. 2011). Many orchid species in Madagascar and elsewhere are in dire need of study to keep pace with projected extinction rates this century. For researchers in distant countries who aspire to study these orchids for conservation purposes, the great distances involved, coupled with limited funds, pose formidable challenges. These challenges are often exacerbated by political unrest, delays in securing permits, unexpected hardships, and the risk that the biological samples collected (e.g., roots harboring mycorrhizal fungi) will not survive long-distance transport. As this study has shown, endophytes of orchids persist in root samples at least 3 weeks after they are collected in the field, as Richardson et al. (1993) reported earlier for epiphytes collected in Costa Rica.

**Fig. 1:**
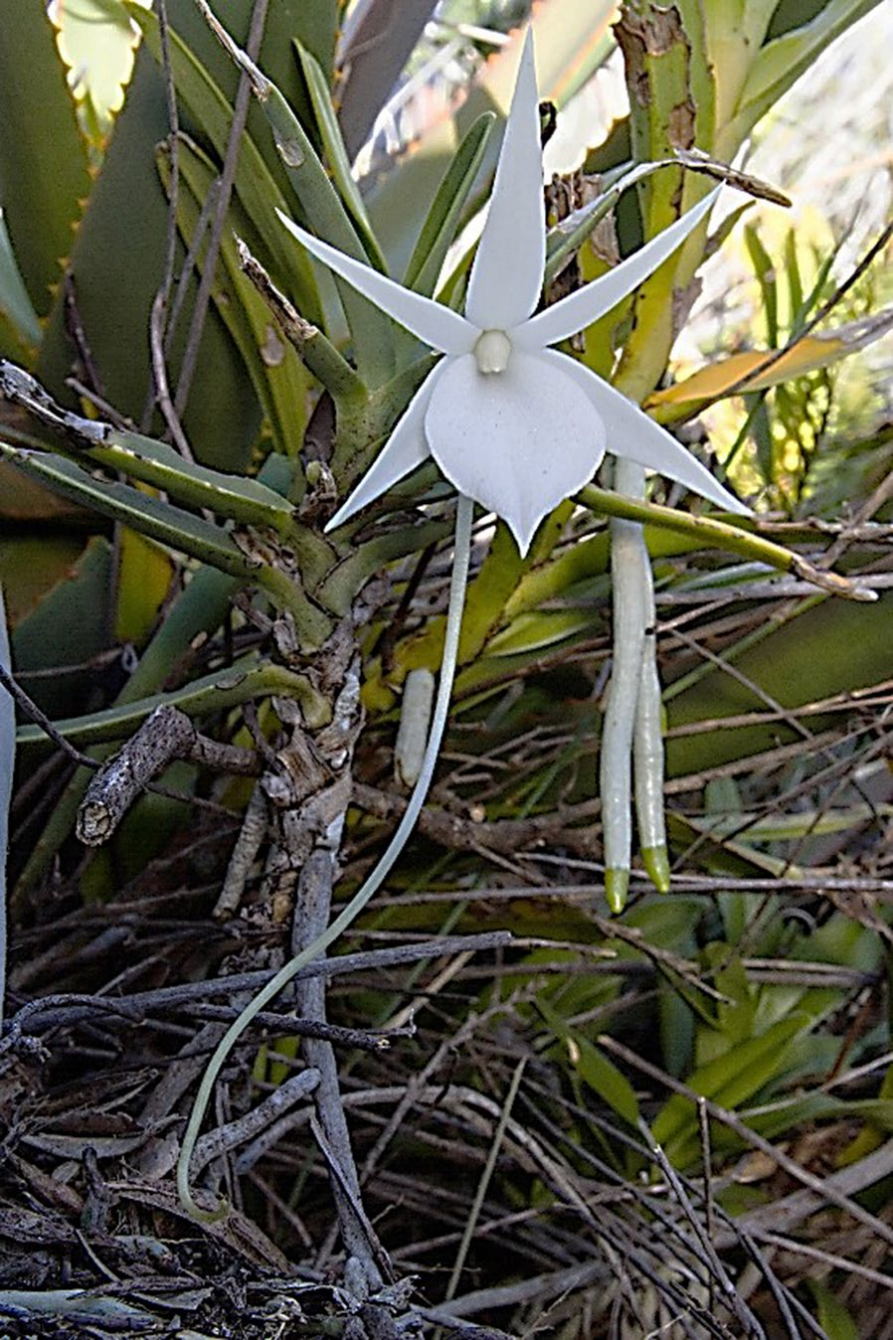
(Left)—*Angraecum protensum*—one of several lithophytic orchids endemic to the Itremo Massif of the Central Highlands in Madagascar, shown in flower during the dry season (June 2012). The long nectar spur is visible to the lower left, and two young roots are seen on the lower right

**Fig. 2:**
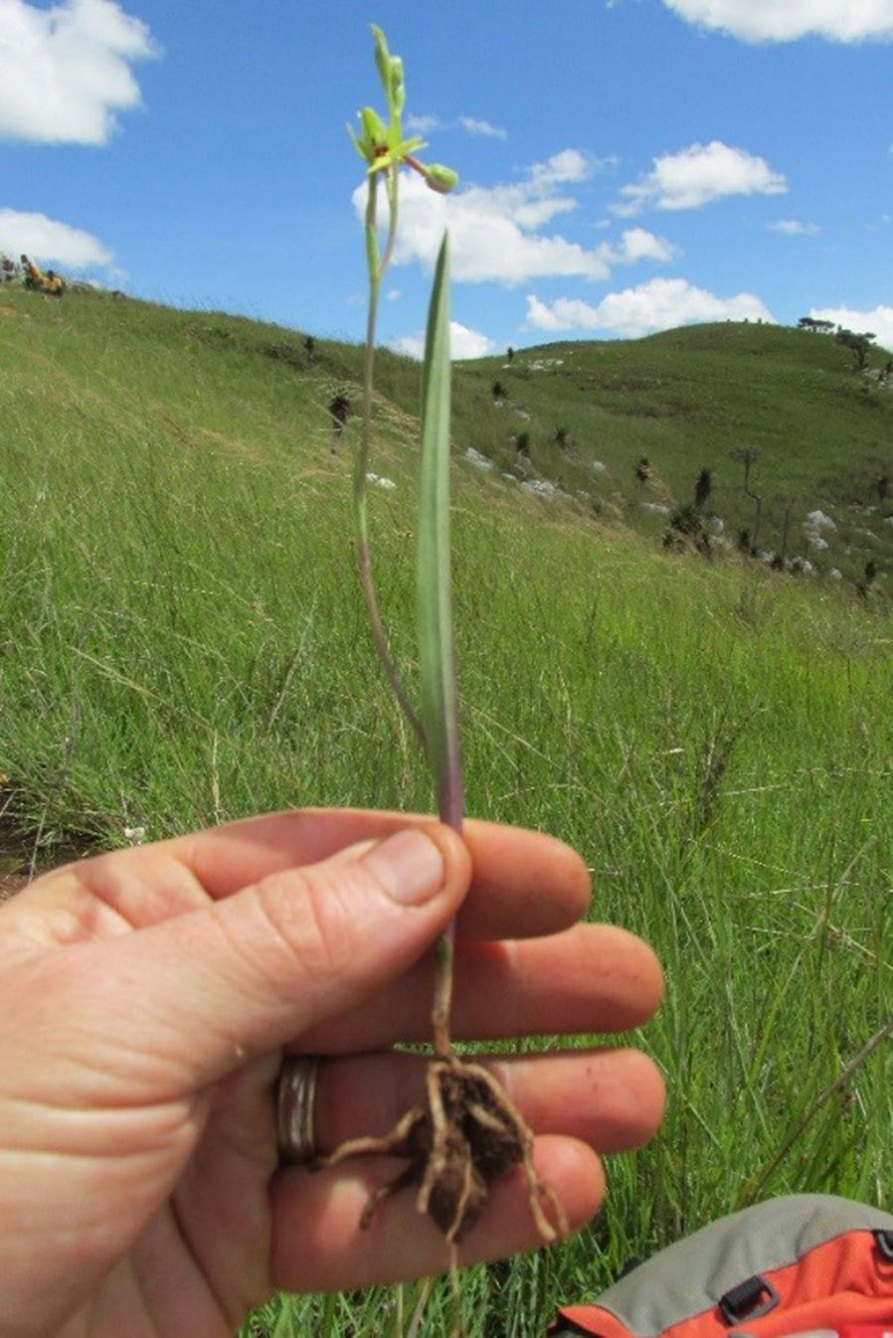
(Right)—The terrestrial orchid, *Cynorkis flexuosa*, shown against the backdrop of open rocky grassland of the Itremo Massif. There are ca 135 species of *Cynorkis* species in Madagascar alone. The lateral branch roots on this specimen were detached and transported to labs in the United States (Illinois) and United Kingdom (Kew) for fungal isolation

In 2012, we were presented with a unique opportunity to collect and study endophytes of rare orchids native to the Itremo Massif Protected Area in the Central Highlands of Madagascar—one of the top five biologically diverse “hotspots” (Tyson 2000). Our primary goal was to isolate, identify, and safeguard *Rhizoctonia*-like fungi to facilitate orchid conservation in the region (e.g., symbiotic germination). The orchids targeted included endemic species (e.g., *Angraecum protensum*, Fig. 1), epiphytes (e.g., *Bulbophyllum*, *Polystachya*), terrestrials (e.g., *Cynorkis flexuosa*, Fig. 2), and lithophytes (e.g., *Angraecum*, *Aerangis*). One prevailing concern, however, was the great distances between our two labs and the Indian Ocean country (Illinois = 15,300 km, London = 9000 km), namely if orchid endophytes would remain viable in living tissues during lengthy, long-distance transport over rugged terrain and by air.

In this paper, we describe our protocol for the collection and long-distance transport of orchid root samples from Madagascar to labs in the United Kingdom (Kew) and the United States (Illinois) leading to isolation and provisional identification of the endophytes. The goal of this paper is to provide other researchers with a workable framework for recovering *Rhizoctonia*-like fungi from aged (2–3 week-old) tissue samples acquired from terrestrial, epiphytic, and lithophytic orchids in remote areas.

## Methods

### Permits

Logistic and taxonomic support for this joint study between Royal Botanic Gardens Kew (Kew) and Illinois College was facilitated by Kew Madagascar Conservation Centre (KMCC) and Parc Botanique et Zoologique de Tsimbazaza (PBZT). During the 5-year project, more than 40 taxa spanning 24 genera were selected for study involving collection and movement of live material (roots, seeds) from Madagascar to labs in the United Kingdom (UK) and the United States (USA). Root samples were shared between the two partners to ensure that live material could be processed in a timely manner leading to the isolation of *Rhizoctonia*-like fungi from root pelotons. To facilitate the legal collection and international transport of orchid material from Madagascar to the UK (Kew) and USA (Illinois), a CITES permit was obtained which allowed three tubes, each containing seedlings and mature roots per species, to be collected. The rarity of plants in the wild necessitated further restrictions, namely that collections be limited to three each of juvenile and mature plants. Depending on the species and its availability, 1–5 roots per specimen were collected. These were accompanied by a phytosanitary certificate which was secured prior to departure from Madagascar. The CITES permit and phytosanitary certificate were delivered by the Direction Generale des Forets (DGF Nanisana Antananarivo) and Service de la Quarantaine et de I’Inspection Nanisana, respectively. For import of root samples into the USA, an additional permit (PPQ 526) was obtained from the US Department of Agriculture (USDA), as the US Government regards all orchid endophytes in the *Rhizoctonia* complex to be plant pathogens. Samples entering the UK were accompanied by a Phytosanitary Certificate issued in Madagascar, and a UK Letter of Authority.

### Dates and study sites in Madagascar

Three separate trips to Madagascar took place spanning 4 years (2012–2015), with emphasis on the collection of roots from mature plants and spontaneous seedlings of lithophytes, epiphytes and terrestrials inhabiting the Itremo Massif Protected Area of the Central Highlands. The first two trips (June 2012, April/May 2013) were planned after the rainy season (December to March) in an effort to collect spontaneous seedlings on orchid-rich substrates that may have germinated under the wetter conditions. The third trip (January 2015) took place during the rainy season to isolate additional fungal strains that may have been inactive during the previous two trips.

### Collection and long-distance transport

**Fig. 3:**
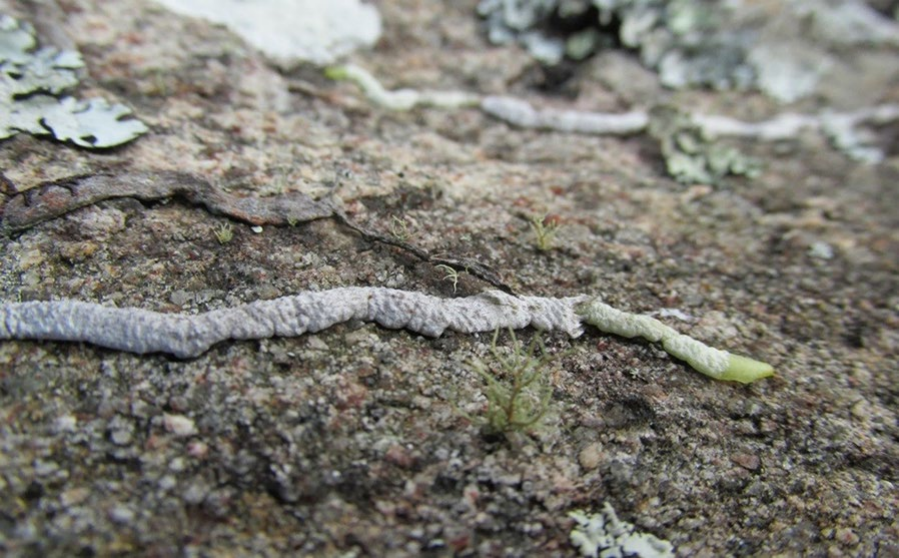
(Bottom)—Actively-growing root tip of a lithophytic *Angraecum* species. Note the green pigmented tip, and root growth on bare rock substrate devoid of visible organic debris, often typical of lithophytes of the Itremo Massif

**Fig. 4:**
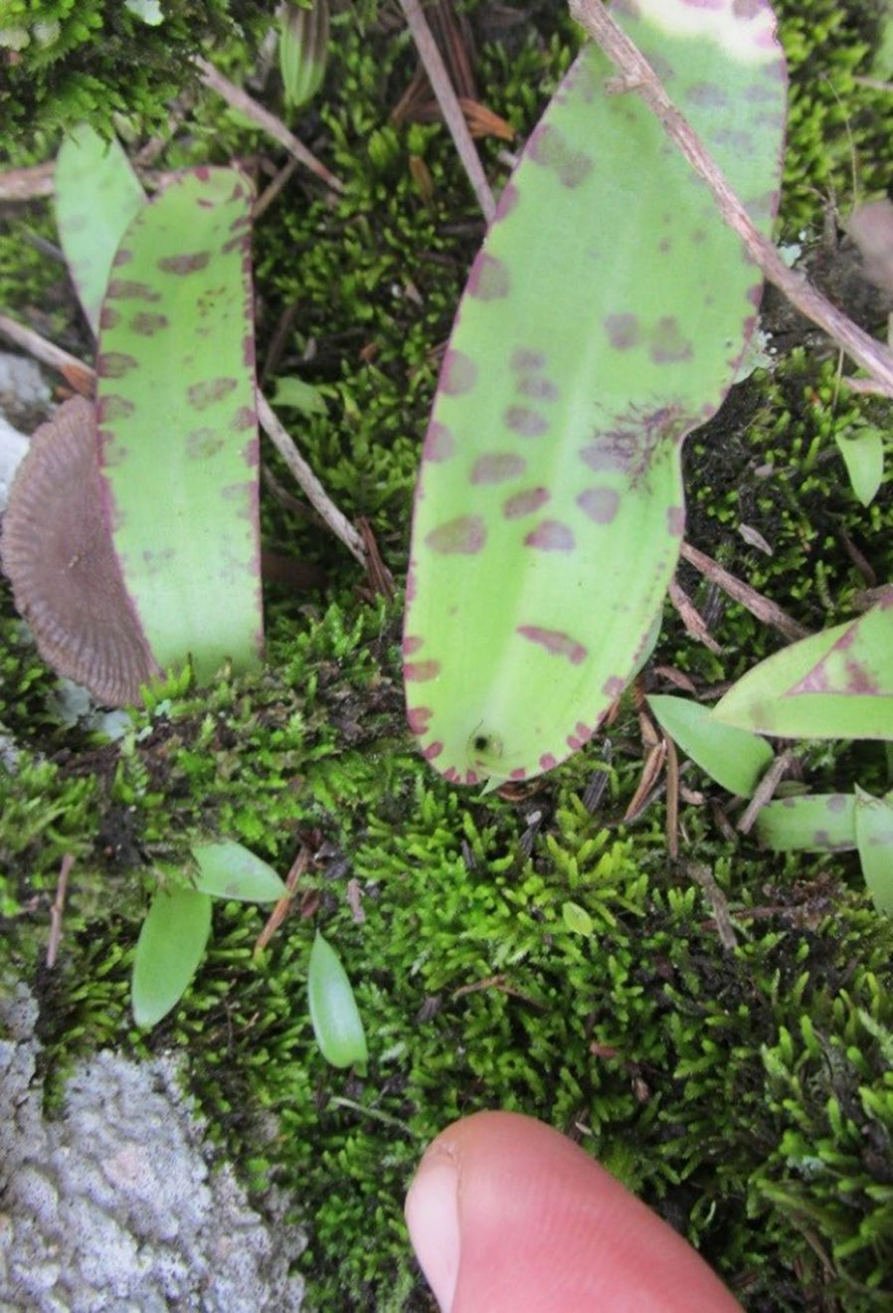
(Left) A strap leaf of the terrestrial orchid, *Cynorkis gibbosa*, showing characteristic mottled pigmentation, rooted in moss on a granite outcrop/seepage slope on the Itremo Massif. Small seedlings are shown at the lower left, presumably from the same species. Seedling stages are often detected in close proximity to mature plants sharing the same substrate

**Fig. 5:**
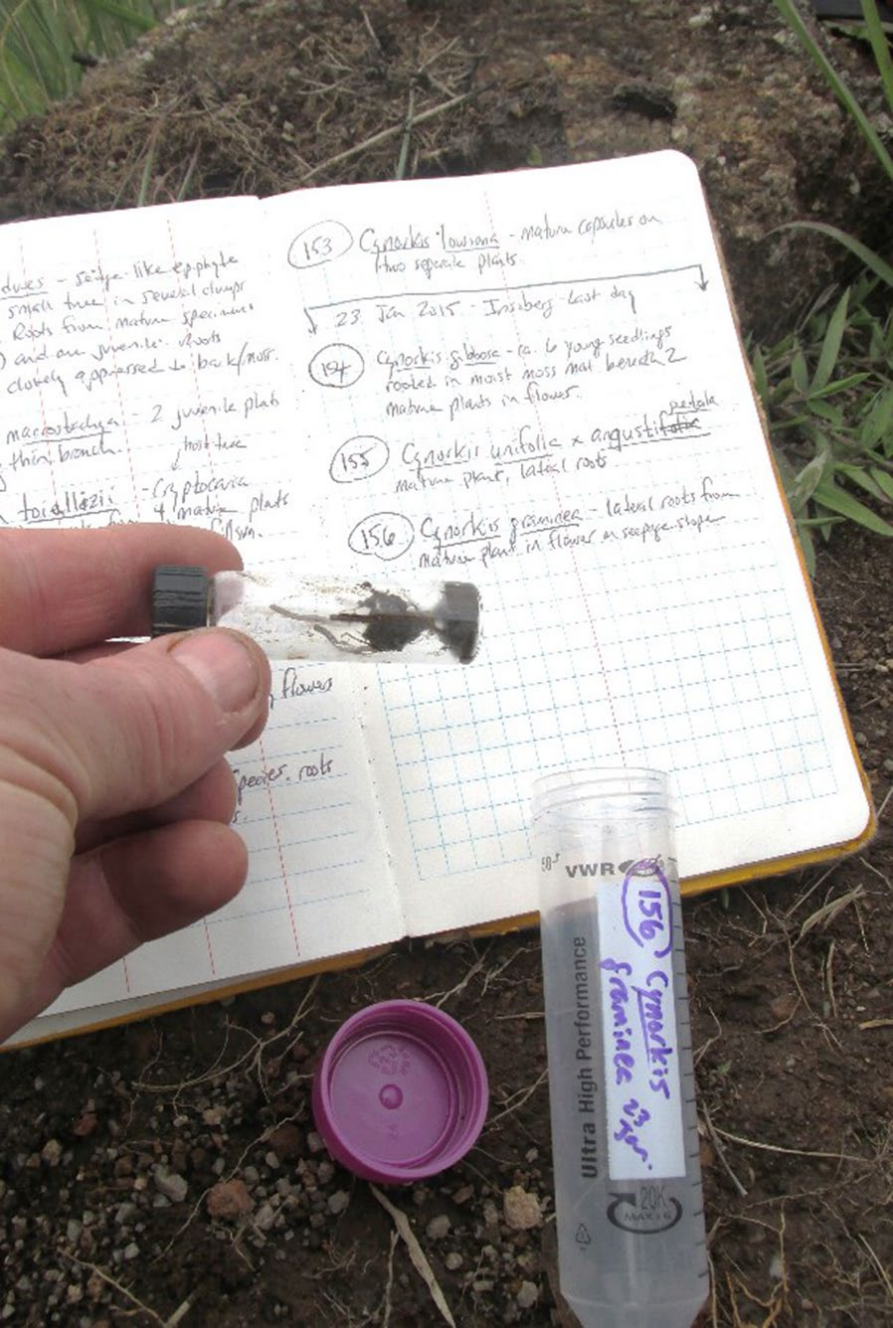
(Right) A pre-sterilized glass sampling vial containing root segments of a terrestrial orchid. The screw cap lid remained untightened to permit gas exchange leading up to air transport out of Madagascar. Each glass vial was placed into a plastic, shatterproof centrifuge vial during transport from Madagascar to the lab

The collection procedure reported by Yokoya et al. (2015) was employed for all three trips to Madagascar, and is summarized below. During the first trip, roots of mature orchids (= those that achieved anthesis or large enough to do so) were primarily targeted for collection to document the location of fungal pelotons in roots, and to isolate fungi in pure culture via hyphal tips. In the two trips that followed, emphasis was placed on collecting roots of seedlings and mature plants alike. Seedlings (= smallest leaf bearing stage, < 2 cm in length) and juveniles (plants > 2 cm in length, no anthesis) were provisionally identified on site by KMCC staff (Rajaovelona and Gardiner 2016) using subtle morphological features (e.g., presence of pseudobulbs) as well as proximity to mature plants on or near the same substrate. The identities of the seedlings were later confirmed by DNA analysis following the procedures described by Yokoya et al. (2015). To maximize our chances for isolating viable pelotons, younger-appearing roots were collected whenever possible. For epiphytic and lithophytic orchids, these roots appeared translucent to white in color, often with slight greenish pigmentation near the apex (Figs. 1 and 3). For terrestrials, roots that exhibited orange-yellowish patches of color were selected, as well as seedlings in close proximity to mature plants (Fig. 4). After detachment in the field, each root was placed over a small, pre-moistened cotton ball within pre-sterilized glass vial with screw cap (Fig. 5). To permit gas exchange leading up to departure from Madagascar (7–10 days after collection), the caps placed on each vial were not securely tightened. The vials were then placed within a 50 ml capacity centrifuge tube with screw cap (VWR International, LLC, Radnor, PA, USA). Tubes were then stored vertically within an insulated handbag for transport from field to shelter. Care was taken to keep the handbag out of direct sunlight so that the root samples would remain as cool as possible (15–25 °C).

For terrestrial orchids, the root collection procedure differed slightly in that soil containing intact root systems (root ball) was also collected. This permitted the roots to remain in a semi-natural (moist) state leading up to departure from Madagascar. A trowel or small shovel was used to gently excavate the soil around individual plants, and to lift the root ball with minimal disturbance to the brittle root systems. Each root ball was then placed into its own separate plastic bag, and the bags were then carefully packed into an insulated handbag for transport. A wet bath towel was then placed through the zip to facilitate wicking and evaporation to the outside air serving to cool the inside of the bag. Upon arrival at the KMCC base in Antananarivo, 2–7 days after field collection, root samples and root balls were placed into a refrigerator (ca. 6 °C). Approximately 24 h before departure from the country, roots of terrestrial orchids were carefully lifted from soil and rinsed off with UV-irradiated and/or bottled water to remove organic debris to comply with US and UK important regulations. Lateral branch roots were detached and placed over a pre-moistened cotton ball in a pre-sterilized glass vial (8 ml capacity). The screw cap was then tightened firmly and wrapped with a strip of Parafilm “M” (Pechiney Plastic Packaging, Menasha, WI, USA. Caps on glass vials containing roots of lithophytes and epiphytes were also tightened and wrapped with Parafilm “M” at that same time (ca. 24 h prior to departure by air). All sealed glass vials were then housed in 50 ml plastic (shatter-proof) centrifuge vials which were also firmly tightened and sealed with Parafilm “M”. Vials were re-packed into insulated handbags and transported back to labs in the USA and UK as cabin luggage to ensure that samples were maintained at ambient temperature during the duration of each flight.

### Fungal isolation and provisional identification

Immediately upon arrival into the UK and USA, within 24 and 48 h after departure from Madagascar, respectively, all root samples were placed in refrigeration (4–6 °C) for a period lasting 1–2 weeks. Fungi were isolated from root cortical regions using the method by Zettler et al. (2003), but our procedure differed in that Fungal Isolation Medium (FIM) substituted for Modified Melin-Norkrans’ agar (MMN). Clumps of macerated cortical cells containing pelotons were immersed in FIM containing streptomycin sulfate [(10 ml/l of stock solution = 1 g dissolved in 70 ml); Clements and Ellyard 1979] and incubated at ambient temperature until actively-growing hyphae could be observed under a dissection microscope (typically 1–4 days). Hyphal tips from cortical cells and/or pelotons were then subcultured to Potato Dextrose Agar (PDA, Difco™, Becton, Dickinson and Co., Sparks, MD, USA) using a sterile scalpel. Provisional identification of *Rhizoctonia*-like fungi to genus level (*Ceratobasidium*, *Sebacina*, *Tulasnella*) reported herein was based on cultural descriptions reported by Currah et al. (1997).

## Results

### Rhizoctonia-like fungi from Madagascar samples

Pelotons were observed in mature roots in half of the lithophytes, half of the epiphytes, and both terrestrials (Table 1). Most of the pelotons were observed in the apex of the roots, in the 1–5 cm region from the tip (Table 1). Four of the seven lithophytic *Angraecum* species harbored pelotons (*A. calceolus*, *A. longicalcar*, *A. magdalenae*, *A. rutenbergianum*; Table 1). Once plated on agar, all pelotons yielded common conidial fungi (*Fusarium* sp., *Trichoderma* sp.), and none resulted in isolates that were assignable to the *Rhizoctonia* complex.

**Table 1:** Peloton location by root region for orchid samples acquired in the Itremo Massif within the Central Highlands of Madagascar during the first of three trips (June 2012, dry season)

Growth habit	Orchid species	*n*	1	2	3	4	5	6	7	8	9	10
Lithophytic	*Angraecum calceolus*	2/2			X	X						X
	*Angraecum longicalcar*	1/3			X							
	*Angraecum magdalenae*	1/2		X								
	*Angraecum obesum*	0/1	–	–	–	–	–	–	–	–	–	–
	*Angraecum protensum*	0/1	–	–	–	–	–	–	–	–	–	–
	*Angraecum sororium*	0/2	–	–	–	–	–	–	–	–	–	–
	*Angraecum rutenbergianum*	1/1	X	X								
	*Jumellea ibityana*	0/2	–	–	–	–	–	–	–	–	–	–
Epiphytic	*Bulbophyllum* sp.	3/3	X		X				X			
	*Bulbophyllum bicoloratum*	0/2	–	–	–	–	–	–	–	–	–	–
	*Jumellea* sp.	1/4	X									
	*Jumellea arborescens*	0/2	–	–	–	–	–	–	–	–	–	–
	*Jumellea intricata*	1/2				X						
	*Polystachya* sp.	0/1	–	–	–	–	–	–	–	–	–	–
	*Polystachya cultriformis*	1/2					X					
	*Polystachya fusiformis*	0/1	–	–	–	–	–	–	–	–	–	–
Terrestrial	*Cynorkis* sp.	1/1		X								
	*Eulophia* sp.	1/2				X						

Numbers 1–10 reflect the distance (in cm) from the root tip (e.g., *1* terminal end of actively-growing tip, *2* second cm region from root tip), and *n* the number of roots harboring pelotons/number of roots collected

Following the second trip to Madagascar (April–May 2013), root samples returned to Illinois yielded *Rhizoctonia*-like fungi in two of the nine epiphytes (*Aerangis* sp., *Polystachya concreta*), both of which were seedlings (Table 2). Of the 14 terrestrial taxa sampled, roots from six species yielded endophytes assignable to the *Rhizoctonia* complex (*Benthamia rostratum*, *Cynorkis purpurea*, *Eulophia macra*, *Graphorkis concolor*, *Habenaria ambositrana*, and *Tylostigma nigrescens*), but none were recovered from the seven lithophytes (Table 2). Collectively, less than half (40%) of the roots of terrestrial orchids spanning all three growth stages (seedlings, juveniles, mature plants) yielded *Rhizoctonia*-like fungi, and 20% for the epiphytes (Table 2). Seedlings of *C. purpurea* harbored the most diverse assemblage of orchid endophytes with of all three major genera being represented in the samples (*Ceratobasidium*, *Tulasnella*, *Sebacina*).

**Table 2:** A summary of the frequency of *Rhizoctonia*-like fungi acquired from roots of orchids inhabiting the Itremo Massif of the Central Highlands of Madagascar during April–May 2013 (dry season)

Growth habit	Orchid	Site	Sample	Fungus (# strains)
Lithophytic	*Angraecum coutrixii*	1, 3	Seedling	None
	*Angraecum longicalcar*	2	Mature	None
	*Angraecum magdalenae*	3	Seedling	None
	*Angraecum protensum*	1	Seedling Mature	None None
	*Angraecum rutenbergianum*	1	Seedling	None
	*Angraecum sororium*	3	Seedling	None
	*Oeceoclades* sp.	2	Mature	None
Epiphytic	*Aerangis* sp.	7	Seedling	*Ceratobasidium* (3)
	*Aerangus citrata*	5	Seedling	None
	*Angraecum* sp.	5	Seedling	None
	*Angraecum protensum*	1	Seedling	None
	*Angraecum rutenbergianum*	3	Seedling	None
	*Bulbophyllum* sp.	3	Seedling	None
	*Jumellea denisfolliata*	5	Seedling	None
	*Polystachya concreta*	1	Seedling 1 Seedling 2	None *Tulasnella* (7), *Sebacina* (6)
	*Polystachya culturiformis*	7	Seedling	None
Terrestrial	*Benthamia* sp.	1	Mature	None
	*Benthamia glaberrima*	3	Mature	None
	*Benthamia rostratum*	4	Juvenile	*Tulasnella* (1)
	*Calanthe* sp.	7	Mature	None
	*Cynorkis gibbosa*	7	Mature	None
	*Cynorkis purpurea*	7	Seedling	*Ceratobasidium* (7) *Tulasnella* (3), *Sebacina* (1)
	*Disa incarnata*	3	Mature	None
	*Eulophia macra*	2	Mature	*Tulasnella callospora* (1)
	*Graphorkis concolor*	7	Mature	*Ceratobasidium* (1)
	*Habenaria* sp.	1	Mature	None
	*Habenaria ambositrana*	1	Juvenile	None
		3	Seedling	*Tulasnella* (4), *sebacina* (1)
	*Satyrium trinerve*	4	Mature	None
	*Tylostigma* sp.	4	Mature	None
	*Tylostigma nigrescens*	4	Seedling	*Tulasnella* (5)

Fungal genera listed represent provisional identifications carried out at the time of isolation, based on cultural characteristics described by Currah et al. (1997). Growth habit reflects the substrate where the individual orchid was actually rooted at the time of collection. Collection sites: *1* exposed rocks, occasional tapia trees, *2* exposed marble outcrop, *3* exposed rocks, sandy stream bed, gnarled small trees, *4* open grassland, moist soil, occasional rocks, *5* reduced forest (canopy ca. 20 m), *6* exposed ridges, montane vegetation, *7* dense shaded forest, downhill stream. With one exception (2), all sites were within 5 km of one another

Terrestrial seedlings = 3/3, epiphytic seedlings = 2/10, lithophytic seedlings = 0/5

Terrestrial juveniles = 1/2, epiphytic juveniles = NA, lithophytic juveniles = NA

Terrestrial mature = 2/10, epiphytic mature = NA, lithophytic mature = 0/3

Total terrestrial = 6/15 (40%), total epiphytic = 2/10 (20%), total lithophytic = 0/8 (0%)

Following the third and final trip to Madagascar coinciding with the rainy season (January 2015), a greater number of *Rhizoctonia*-like fungi were recovered from root samples collected 2–3 weeks prior, including a lithophyte, *Eulophia* sp. (Table 3). Among the six epiphytic taxa sampled, seedlings from *Aerangis* and *Polystachya* yielded *Ceratobasidium* and *Tulasnella* strains, respectively (Table 3). The majority of fungal isolates were acquired from terrestrials, in particular *Cynorkis* species (Table 3).

**Table 3:** A summary of the frequency of *Rhizoctonia*-like fungi acquired from roots of orchids inhabiting the Itremo Massif of the Central Highlands of Madagascar during January 2015 (rainy season)

Growth habit	Orchid	Site	Sample	# Strains and fungus
Lithophytic	*Eulophia* sp. 1	1	Seedling	None
	*Eulophia* sp. 1	1	Mature	*Tulasnella* (1)
	*Jumellea densifolia*	2	Seedling	None
Epiphytic	*Aerangis* sp. 1	5	Seedling	*Ceratobasidium* (10)
	*Angraecum* sp. 1	2	Seedling, juvenile	None
	*Angraecum* sp. 3	6	Mature	None
	*Aeranthes* sp. 1	6	Juvenile, mature	None
	*Bulbophyllum baronii*	2	Mature	None
	*Polystachya* sp. 1	6	Seedling	*Tulasnella* (4)
	*Polystachya concreta*	3	Seedling	None
Terrestrial	*Angraecum* sp. 2	2	Mature	None
	*Benthamia* sp. 1	3	Mature	None
	*Calanthe sylvatica*	5	Mature	None
	*Cynorkis* sp. 1	3	Mature	*Ceratobasidium* (2)
	*Cynorkis* sp. 1	4	Mature	*Tulasnella*, *sebacina* (2)
	*Cynorkis* sp. 1	7	Mature	None
	*Cynorkis fastigiata*	4	Mature	*Ceratobasidium* (1)
	*Cynorkis fastigiata*	6	Mature	*Tulasnella* (3)
	*Cynorkis flexuosa*	1	Mature	None
	*Cynorkis flexuosa*	1	Seedling	*Tulasnella* (2)
	*Cynorkis gibbosa*	4	Mature	*Tulasnella* (4)
	*Cynorkis gibbosa*	4	Juvenile	None
	*Cynorkis gibbosa*	7	Seedling	None
	*Cynorkis uniflora*	3	Seedling	*Ceratobasidium* (1)
	*Cynorkis uniflora*	3	Mature	None
	*Cynorkis uniflora*	7	Mature	None
	*Disa* sp. 1	4	Mature	None
	*Eulophia* sp. 1	1	Seedling	None
	*Eulophia plataginea*	1	Juvenile	None
	*Habenaria* sp.1	1	Juvenile, mature	None
	*Habenaria* sp. 2	2	Seedling	*Tulasnella* (1)
	*Habenaria* sp. 2	4	Mature	*Tulasnella* (1)
	*Jumellea* sp.	1	Mature	None
	*Liparis* sp. 1	5	Seedling	*Tulasnella* (1)
	*Polystachya* sp. 1	2	Mature	None
	*Satyrium* sp.	5	Seedling	*Tulasnella* (1)
	*Satyrium trinerve*	3	Mature	None
	*Satyrium trinerve*	4	Seedling	*Tulasnella* (4)

Fungal genera listed represent provisional identifications carried out at the time of isolation, based on cultural characteristics described by Currah et al. (1997). Growth habit reflects the substrate where the individual orchid was actually rooted at the time of collection. Collection sites: *1* abandoned mine, rocky grassland, *2* tapia forest, *3* seepage slope, *4* grassland, seepage slope, *5* forest preserve, *6* forest, *7* rocky elevated grassland

Terrestrial seedlings = 6/8, epiphytic seedlings = 2/4, lithophytic seedlings = 0/2

Terrestrial juveniles = 0/3, epiphytic juveniles = 0/2, lithophytic juveniles = NA

Terrestrial mature = 6/18, epiphytic mature = 1/4, lithophytic mature = 1/1

Total terrestrial = 12/29 (41%), total epiphytic = 3/10 (30%), total lithophytic = 1/3 (33%)

## Discussion

As to why none of the samples from the first trip yielded *Rhizoctonia*-like fungi is perplexing, but might be attributed, in part, to the time of year of the collecting. For example, sampling during the first trip took place in June, whereas the second took place earlier in the year (April/May) closer to the end of the rainy season. Thus, it is conceivable that the samples collected in June were devoid of *Rhizoctonia*-like fungi because of drier conditions. The two subsequent trips that ensued yielded endophytes assignable to all three major genera of orchid mycorrhizal fungi (*Ceratobasidium*, *Tulasnella*, *Sebacina*). For roots of epiphytic and lithophytic orchids, placing detached roots into pre-sterilized vials that remain unsealed possibly served to allow gas exchange (oxygen) for roots and endophytes alike. Moist sterile cotton balls within the vials also may have maintained higher relative humidity levels needed for root and fungus longevity. For terrestrial orchids, keeping roots intact within the moist soil/root ball may have contributed to the high number of *Rhizoctonia*-like fungi acquired from the samples. Placing the vials in an insulated bag during field work, and temporary storage in refrigeration whenever possible, may have also benefited endophyte survival by maintaining cooler temperatures, slowing down metabolic rates in living cells.

Root colonization by endophytic fungi is thought to be influenced by two important factors—the growing season, and the growth stage of the plant (Swarts and Dixon 2017). For temperate terrestrial orchids, Harvais and Raitsakas (1975) and Warcup (1973) found that the most effective fungi for the purposes of seed germination were acquired earlier in the growing season. Huynh et al. (2004) reported that fungi isolated from pelotons in early growth stages were most effective at facilitating seed germination. A prevailing concern we had for collecting smaller roots of seedlings and juvenile stages during the dry season (April/May 2013) was dehydration of the samples given their high surface-to-volume ratio. This potential problem was apparently avoided by the addition of a moist (sterile) cotton ball placed inside the vial with the sample, as several of *Rhizoctonia*-like isolates were later recovered. Some of these isolates were later tested for their ability to germinate seeds in vitro, with positive results. For example, one of the six strains of *Sebacina*, isolated from a *P. concreta* seedling, was most effective among 14 endophytes tested at inducing rapid in vitro seedling development of *C. purpurea* in symbiotic germination studies that ensued (Rafter et al. 2016). In another experiment, seeds of *H. ambositrana* and *T. nigrescens*, yielded leaf-bearing seedlings in vitro, 49 days after inoculation with *Sebacina* and *Tulasnella* endophytes acquired from seedlings of the same species, respectively (A. Wood, unpub. data).

To what extent mature epiphytic orchids harbor and utilize *Rhizoctonia*-like fungi in Madagascar needs further study. Due to permit restrictions, only a select few taxa could be collected the third and final year of the study, and the decision was made to target orchid tissues that were most likely to harbor *Rhizoctonia*-like fungi, namely seedlings. Roots collected from the lone mature epiphyte (*Bulbophyllum baronii*) were devoid of pelotons, and therefore no endophytes were isolated (Table 3). For future work on the Itremo Massif and the Central Highlands in general, we recommend that roots of mature epiphytes be collected during the rainy season (January). For epiphytic orchids occupying Madagascar’s eastern and northern forests where rainfall is more plentiful throughout the year (> 2000 mm annually; Cribb and Hermans 2009), time of collection may be less critical, as higher moisture levels would be expected to favor fungal activity. To our knowledge, no studies have been reported that document *Rhizoctonia*-like fungi from Orchidaceae inhabiting the NE portion of the island. Given Madagascar’s considerable biodiversity, securing fungi from both areas and comparing the orchid mycoflora between the two regions seems like a logical next step.

As a group, roots of mature lithophytic orchids yielded relatively few *Rhizoctonia*-like fungi with one exception (*Tulasnella* from *Eulophia* sp. 1, Table 3). Mature roots from six lithophytes collected the first year harbored pelotons primarily in the first 4 cm of the root tip (Table 1), yet these pelotons yielded conidial saprophytes (*Fusarium, Trichoderma*), not typical genera in the *Rhizoctonia* complex. As to why this occurred is puzzling and deserves further inquiry. It is conceivable that roots of these lithophytes were subjected to rapid dehydration given their dry placement on sun-exposed rocks with little or no associated (moist) organic debris (Fig. 3). Thus, the pelotons we observed in the tissues may have been formed by *Rhizoctonia*-like fungi during the rainy season, but quickly dried out before the pelotons could be completely digested by the orchid (lysis). Opportunistic saprophytes may then have gained entry as secondary invaders, which may explain why we isolated *Fusarium* in the present study, as well as slow-growing, dark-pigmented endophytes (*Toxicocladosporium*, *Cladophialophora*, *Lophiostoma*) recovered in samples at Kew (Yokoya et al. 2015). Studies are needed to explore the true nature of the peloton-forming, non-*Rhizoctonia* fungi to determine if these endophytes are potentially harmful and/or benign inhabitants, or if they serve a physiological purpose.

Moisture availability (retention) linked with seasonality may also explain why more *Rhizoctonia*-like fungi were isolated from epiphytes compared to lithophytes. For example, roots of many of the epiphytes we sampled were tightly affixed to crevices of tree bark often in association with lichens and mosses. All three of these substrates would be expected to retain water more effectively than bare rock alone, and could also serve as a carbon source for associated fungi, including the *Rhizoctonia*-like fungi present within the orchid. Members of *Ceratobasidium*, in particular, are known to produce polyphenoloxidases that are involved with lignin breakdown (Rasmussen 1995), and this may explain why roots of some epiphytes, namely *Aerangis*, harbored mostly *Ceratobasidium* (Tables 2 and 3). Thus, the utilization of *Ceratobasidium* strains may afford epiphytes like *Aerangis* with an additional source of organic carbon, and therefore a selective advantage for life in the canopy. For lithophytes, utilization of *Ceratobasidium* at an early (protocorm) stage of development may be critical to life on the rocks in pockets where organic debris and moisture accumulate.

## Conclusions

Despite the distance, rugged terrain, and length of time between field collection and transport to the laboratory, roots of epiphytic, lithophytic and terrestrial orchids yielded all major groups of fungi in the *Rhizoctonia* complex (*Ceratobasidium*, *Tulasnella*, *Sebacina*). These fungi were present in roots of seedlings, juveniles and mature plants, especially terrestrials. Despite their small size, root pieces from seedlings stages of epiphytes (e.g., *P. concreta*) yielded fungi with our method despite being detached in the field 3 weeks prior. In cases where peloton extraction cannot be accomplished in a timely manner (1–4 days of collection), our study demonstrates that samples retain viability up to 3 weeks after collection.

## References

[CR1] Aggarwal S, Nirmala C, Beri S, Rastogi S, Adholeya A (2012). In vitro symbiotic seed germination and molecular characterization of associated endophytic fungi in a commercially important and endangered Indian orchid, *Vanda coerulea* Griff. ex Lindl. Eur J Environ Sci.

[CR2] Chen J, Wang H, Guo SX (2012). Isolation and identification of endophytic and mycorrhizal fungi from seeds and roots of *Dendrobium* (Orchidaceae). Mycorrhiza.

[CR3] Chutima R, Dell B, Vessabutr S, Bussaban B, Lumyong S (2011). Endophytic fungi from *Pecteilis susannae* (L.) Rafin (Orchidaceae), a threatened terrestrial orchid in Thailand. Mycorrhiza.

[CR4] Clements MA, Ellyard RK (1979). The symbiotic germination of Australian terrestrial orchids. Am Orchid Soc Bull.

[CR5] Cribb PJ, Hermans J (2009). Field guide to the orchids of Madagascar.

[CR6] Cribb PJ, Kell SP, Dixon KW, Barrett RL (2003) Orchid conservation: a global perspective. (Chapter 1, pp 1–24). Mycorrhizal diversity (Chapter 11, pp 185–203). In: Dixon KW, Kell SP, Barrett RL, Cribb PJ (eds) Orchid conservation. Natural History Publications, Kota Kinabalu, pp 205–226 (ISBN 983-812-078-2)

[CR7] Currah RS, Sigler L, Hambleton S (1987). New records and new taxa of fungi from the mycorrhizae of terrestrial orchids of Alberta. Can J Bot.

[CR8] Currah RS, Zelmer CD, Hambleton S, Richardson KA, Arditti J, Pridgeon AM (1997). Fungi from orchid mycorrhizas. Orchid biology: reviews and perspectives, VII.

[CR9] Dressler RL (1993). Phylogeny and classification of the orchid family.

[CR10] Harvais G, Raitsakas A (1975). On the physiology of a fungus symbiotic with orchids. Can J Bot.

[CR11] Hoang NH, Kane ME, Radcliffe EN, Zettler LW, Richardson LW (2017). Comparative seed germination and seedling development of the Ghost Orchid, *Dendrophylax lindenii* (Orchidaceae), and molecular identification of its mycorrhizal fungus from south Florida. Ann Bot.

[CR12] Huynh TT, McLean CB, Coates F, Lawrie AC (2004). Effect of developmental stage and peloton morphology on success in isolation of mycorrhizal fungi in *Caladenia formosa* (Orchidaceae). Aust J Bot.

[CR13] Jacquemyn H, Brys R, Merckx VS, Waud M, Lievens B, Wiegand T (2014). Coexisting orchids species have distinct mycorrhizal communities and display strong spatial segregation. New Phytol.

[CR14] McCormick MK, Taylor DL, Juhaszova K, Burnett RK, Whigham DF, O’Neill JP (2012). Limitations on orchid recruitment: not a simple picture. Mol Ecol.

[CR15] Merritt DJ, Hay FR, Swarts ND, Sommerville KD, Dixon KW (2014). Ex situ conservation and cryopreservation of orchid germplasm. Int J Plant Sci.

[CR16] Moore-Landecker E (1996). Fundamentals of the fungi.

[CR17] Nontachaiyapoom S, Sasirat S, Manoch L (2010). Isolation and identification of *Rhizoctonia*-like fungi from roots of three orchid genera, *Paphiopedilum*, *Dendrobium*, and *Cymbidium*, collected in Chaing Rai and Chiang Mai provinces of Thailand. Mycorrhiza.

[CR18] Otero JT, Ackerman JD, Bayman P (2002). Diversity and host specificity of endophytic *Rhizoctonia*-like fungi from tropical orchids. Am J Bot.

[CR19] Pereira OL, Rollemberg CL, Borges AC, Matsouka K, Kasuya MCM (2003). *Epulorhiza epiphytica* sp. nov. isolated from mycorrhizal roots in Brazil. Mycoscience.

[CR20] Pereira OL, Kasuya MCM, Borges AC, de Araújo EF (2005). Morphological and molecular characterization of mycorrhizal fungi isolated from neotropical orchids in Brazil. Can J Bot.

[CR21] Rafter M, Yokoya K, Schofield EJ, Zettler LW, Sarasan V (2016). Non-specific symbiotic germination of *Cynorkis purpurea* (Thouars) Kraezl., a habitat-specific terrestrial orchid from the Central Highlands of Madagascar. Mycorrhiza.

[CR22] Rajaovelona L, Gardiner LM (2016). *Angraecum longicalcar*: saving a critically endangered orchid. Orchid Rev.

[CR23] Rasmussen HN (1995). Terrestrial orchids, from seed to mycotrophic plant.

[CR24] Rasmussen HN, Rasmussen FN (2009). Orchid mycorrhiza: implications of a mycophagous life style. Oikos.

[CR25] Richardson KA, Currah RS, Hambleton S (1993). Basidiomycetous endophytes from the roots of neotropical epiphytic Orchidaceae. Lindleyana.

[CR26] Suárez JP, Weiß M, Abele A, Garnica S, Oberwinkler F, Kottke I (2006). Diverse tulasnelloid fungi form mycorrhizas with epiphytic orchids in the Andean cloud forest. Mycol Res.

[CR27] Swarts ND, Dixon KW (2009). Terrestrial orchid conservation in the age of extinction. Ann Bot.

[CR28] Swarts ND, Dixon KW (2017). Conservation methods for terrestrial orchids.

[CR29] Swarts ND, Sinclair EA, Francis A, Dixon KW (2010). Ecological specialization in mycorrhizal symbiosis leads to rarity in an endangered orchid. Mol Ecol.

[CR30] Tyson P (2000). The eighth continent: life, death and discovery in the lost world of Madagascar.

[CR31] Warcup JH (1973). Symbiotic germination of some Australian terrestrial orchids. New Phytol.

[CR32] Warcup JH (1981). The mycorrhizal relationships of Australian orchids. New Phytol.

[CR33] Whitman M, Medler M, Randriamanindry JJ, Rabakonandrianina E (2011). Conservation of Madagascar’s granite outcrop orchids: the influence of fire and moisture. Lankesteriana Int J Orchidol.

[CR34] Yokoya K, Zettler LW, Kendon JP, Bidartondo M, Stice AL, Skarha S, Corey LL, Knight A, Sarasan V (2015). Preliminary findings on identification of mycorrhizal fungi from diverse orchids in the Central Highlands of Madagascar. Mycorrhiza.

[CR35] Zelmer CD, Cuthbertson L, Currah RS (1996). Fungi associated with terrestrial orchid mycorrhizas, seeds and protocorms. Mycoscience.

[CR36] Zettler LW, Sharma J, Rasmussen F (2003) Mycorrhizal diversity (Chapter 11, pp 185–203). In: Dixon KW, Kell SP, Barrett RL, Cribb PJ (eds) Orchid conservation. Natural History Publications, Kota Kinabalu, pp 205–226. (ISBN 983-812-078-2)

[CR37] Zettler LW, Wood EM, Johnson JAN, Kirk AK, Perlman SP (2011). Seed propagation and reintroduction of the US federally endangered Hawaiian endemic, *Platanthera holochila* (Hbd.) Krzl. (Orchidaceae). Eur J Environ Sci.

